# Postoperative Weight Gain, Due to Fluid Retention After Ovarian Cancer Surgery—How Much Is too Much?

**DOI:** 10.1002/jso.70231

**Published:** 2026-03-17

**Authors:** Eva K. Egger, Melisa Delen, Hanno Matthaei, Laura Tascon Padron, Carolin Schröder, Milka Marinova, Dominique Koensgen, Alexander Mustea

**Affiliations:** ^1^ Department of Gynecology and Gynecologic Oncology University of Bonn, University hospital of Bonn Bonn Germany; ^2^ Department of General, Thoracic and Vascular Surgery University of Bonn, University Hospital of Bonn Bonn Germany; ^3^ Department of Nuclear Medicine University of Bonn, University Hospital of Bonn Bonn Germany

**Keywords:** anastomotic leak, complications, fluid management, ovarian cancer, weight gain

## Abstract

**Background and Objectives:**

Postoperative complications after cytoreductive surgery in ovarian cancer patients are associated with impaired survival. Here, we investigated the association between postoperative weight gain due to fluid retention and the development of complications and anastomotic leakage (AL).

**Methods:**

*N* = 278 patients underwent cytoreductive surgery at the university hospital of Bonn in between 01/2013 and 03/2022. Postoperative weight gain on day 2 and day 5 was assessed and correlated to the occurrence of complications according to the MSKCC secondary event score and ALs.

**Results:**

There were 227 surgeries (81.7%) for primary and 51 surgeries (18.3%) for recurrent ovarian cancer. Severe complications were significantly more frequent in patients with postoperative weight gain exceeding 4 kg on postoperative day 2 (26/90 vs. 13/76; *p* = 0.03) and exceeding 3 kg on postoperative day 5 (34/89 vs 30/134; *p* = 0.02). A weight gain of more than 6 kg on postoperative day 2 was significantly associated with the occurrence of AL (*p* = 0.021). Less weight gain on d2 and d5 was associated with a significant earlier first defecation (day‐2 *p*‐value: 0.01; day‐5 *p*‐value: 0.01). In multiple binary logistic regression analysis, the postoperative weight gain on day 2 (*p* = 0.0019) and the performance of an anastomosis (*p* = 0.0095) remained the only significant risk factors regarding severe complications.

**Conclusions:**

Postoperative weight gain, as a surrogate for fluid retention, and the creation of an anastomosis were associated with an increased risk of severe postoperative complications.

## 1 Introduction

Patients with epithelial ovarian cancer face the poorest survival rates of all gynecologic malignancies as of today [1]. Complete cytoreduction with the goal of no residual macroscopic disease is the key to improve progression free and overall survival in ovarian cancer patients [2, 3]. Most patients present in advanced tumor stages with extensive peritoneal tumor spread and need dedicated extensive surgery with high surgical complexity to achieve complete cytoreduction. This harbors the risk of postoperative complications [4]. Complications and subsequent delays in chemotherapy application impair survival [5, 6]. While risk factors for postoperative complications—such as comorbidities, surgical complexity, frailty, tumor burden and duration of surgery—are known [4, 7, 8], there are still understudied, potentially modifiable risk factors to ensure faster convalescence.

One interesting modifiable risk factor is the intra‐ and the postoperative fluid management in ovarian cancer patients. In fact, in colonic cancer patients, randomized trials proofed that restricted perioperative fluids decrease complications and anastomotic leakage [9, 10, 11]. Therefore, the Enhanced Recovery After Surgery Society (ERAS®) recommends to aim for a near to zero balance and restricts the postoperative weight gain due to fluid retention to no more than 2.5 kg [10, 12]. Furthermore, we recently demonstrated that intraoperative fluid management in ovarian cancer patients represents a modifiable risk factor [7].

Nevertheless, maintaining hemodynamic stability in ovarian cancer represents a significant challenge and differs from colonic cancer patients [13]. Median perioperative weight gains of more than 7 kg are reported after cytoreductive surgery [14].

On the basis of these findings, our present study addresses the definition of specific cut‐off values for postoperative weight gain after cytoreductive surgery for ovarian cancer. These may serve as benchmark values to modify fluid management daily to prevent complications and anastomotic leaks.

## 2 Materials and Methods

### 2.1 Data Collection

This observational retrospective cohort study was conducted in accordance with the standards of the ethics committee of the Faculty of Medicine at the University of Bonn, Germany (Nr: 14/22). The institutional database was reviewed identifying patients who underwent cytoreductive surgery for ovarian cancer between January 2013 and March 2022 at the University Hospital of Bonn. Informed consent for the use of the data was obtained within the framework of our biobank initiative.

### 2.2 Patients and Variables

Patients' charts, anesthesiologic protocols, surgery reports and pathologic findings were screened for patient characteristics regarding the preoperative morbidity, expressed by the American Society of Anesthesiologists (ASA)‐Score, time of surgery (upfront or interval surgery after neoadjuvant chemotherapy/surgery for primary disease or recurrence), International Federation of Gynecology and Obstetrics (FIGO) stage, performance of anastomoses, pre‐ and postoperative hemoglobin and hematocrit levels. According to the surgical report, the surgical complexity score [15] and tumor load expressed by peritoneal cancer index (PCI) was calculated for each patient [16]. Furthermore, we reviewed each patient's chart for daily body weight, the volume of intravenous (i.v.) fluids administered during surgery, the day of first defecation, the use of bowel preparation agents and bowel stimulation agents and the use of nasogastric tubes.

The following ERAS recommendations were followed in all patients: a peridural catheter was placed whenever possible, immediate first mobilization on the day of surgery whenever possible, but the latest by the first postoperative day guided by the nursing staff. Immediate removal of the urine catheter as soon as patient's mobilization allowed independent use of the toilet. The first intake of fluids started at 6 h after surgery. In case of anastomoses, fluid oral intake was recommended until the first defecation. Nasogastric tubes were removed at the end of surgery and central venous catheters were removed at the latest by the third day after surgery. All patients received 10 mg Metoclopramide i.v. 3 times a day postoperatively until first defecation. Immediately before the start of surgery and following every 4 h all patients received antibiotics (either 1,5 g cefuroxime and 500 mg metronidazole or 900 mg clindamycin and 500 mg metronidazole). In case of anastomosis patients received antibiotics for a further 3 days at least. Preoperative bowel preparation changed in 2019 from 2000ml Delcoprep® or 2000 ml Oralav® to the sole use of an enema with or without 45 ml sodium phosphate.

### 2.3 Outcome Variables

The primary outcome variable regarding safety was severe postoperative complications within the first 30 days after surgery, graded according to the Memorial Sloan Kettering Cancer Center secondary surgical event score (grade 1 to grade 5; G1‐G5) [17]. Further outcome variables were the incidence of anastomotic leakage (AL) and the time to first postoperative defecation. AL was defined in case of stool in the target drainage or by an extravasation of water‐soluble contrast agent visible on abdominal CT scan. The comparator arm were patients with “no complications and mild complications (G0‐G2)” versus patients with severe complications (G3‐G5) respectively patients with “anastomotic leak” versus “no anastomotic leak” in case of performing an anastomosis.

The variable to assess the postoperative fluid balance was the difference in weight between baseline (the day before surgery) and the second (d2) and fifth (d5) postoperative day. These represent the days with the most complete data available.

*Statistical analysis:* Descriptive statistics of variables are expressed as numbers, proportions, and medians as appropriate. Hypothesis testing for differences between two samples was performed using the Fisher test regarding the outcome. Differences were significant at a threshold of ≤ 0.05. The *t*‐test was used to compare the means between two samples on a 95%‐level. Correlations were tested with the coefficient of determination *R* ^2^. Differences were considered significant at a R² > 80%. To detect significant risk factors for the variable “severe complications“ and the variable “occurrence of an anastomotic leakage” separate multiple binary logistic regression analyses were performed. Multiple binary logistic regression analysis was conducted using block wise entry. The reference groups for the logistic regression model were patients with “no or mild complications” and patients with “no anastomotic leak” respectively. All statistical data analysis was conducted using R (version 4.5.1.).

## 3 Results

*General characteristics*: Between January 2013 and March 2022 there were 278 cytoreductive surgeries for epithelial ovarian cancer in our department. Table 1 shows general patient characteristics and the number of patients where data could be retrieved out of patients' charts. 52 patients were FIGO Stage I (Ia: 25, Ib: 4, Ic1: 4, Ic2: 14, Ic3: 35), 10 patients were FIGO Stage II (IIa: 1, IIB: 9), 189 patients were FIGO Stage III (IIIA1i: 9, IIIA1ii: 4, IIIA2: 2, III B: 22, IIIC: 151), 27 patients were FIGO Stage IV (IVA: 13, IVB: 14) according to the actual FIGO‐Staging [18]. Table 2 depicts the number of patients with an anastomosis and type of anastomosis. 137 (49,3%) patients received a bowel resection with at least one anastomosis. 105 patients received one anastomosis, 31 patients received 2 anastomoses, and 1 patient received 3 anastomoses. There were 169 anastomoses in total and 20 anastomotic leakages (11,8%). All 32 patients with multiple anastomoses received a prophylactic stoma. Further 4 patients received a stoma with only one anastomosis. Table 3 shows the preoperative bowel preparation and the postoperative bowel stimulation. In 2019 a polyethylenglycol (PEG) containing mechanical bowel preparation with Delcoprep® and Oralav® was almost entirely abandoned. In detail, 129 patients out of 167 patients received bowel preparation with PEG from 2013 until 2018. Only 16 out of 111 patients received mechanical bowel preparation with PEG from 2019 until 2022. In case of PEG containing bowel preparation, a postoperative fluid excess (PFE) of more than 5 liters was observed significantly more often (*p*‐value: 0.00). In detail, only 37 (25,3%) out of 146 patients treated with PEG containing bowel preparations had a PFE below 5 liters. In case of other bowel preparations (enema, 45 ml sodium phosphate) the PFE was below 5 liters in 89 (80,2%) out of 127 patients.

**Table 1:** General patient characteristics.

Patient characteristic	Number and percent of patients or median as most appropriate	Range	Number of patiens with data available
Age	Median: 62	16−86 years	278
Postoperative ph.	Median: 7.34	7.12−7.53	278
Postoperative lactate	Median: 1.17	0.32−8.60	237
Duration of surgery	Median: 342	72−695 min	278
PCI	Median: 9	0–28	278
ASA Score	Median: 2 1: 22 2: 164 3: 90 4: 2	1–4	278
Peridural catheter	Yes: 238	n.a.	278
	No: 40		
Need of intensive care	Median: 1 day	1–50 days	278
Length of stay	Median: 15 days	3–197	277
Age adjusted Charlston Comorbidity Score	Median: 2	0–10	278
4‐quadrant ascites present	60 (21.6%)	n.a.	278
No ascites	218 (78.4%)	n.a.	278
Surgery for primary disease	227 (81.7%)	n.a.	278
Surgery for recurrent disease	51 (18.3%)	n.a.	278
Neoadjuvant chemotherapy	81 (29.1%)	n.a.	278
Smoking	Yes: 67 (24.1%); No: 209 (75.2%)	n.a.	276
Surgical complexity score	1: 61 (21.9%); 2: 104 (37.4%); 3: 113 (40.6%)	n.a.	278
Residual tumor at the end of surgery	None: 200 (71.9%); 1 mm: 1 (0.4%); < 5 mm: 35 (12.6%); > 1 cm: 42 (15.1%)	n.a.	278
Transfusion of erythrocyte concentrates (EC)	Median: 0; 127 received ECs (45.7%)	0–14	277
Trasfusion of fresh frozen plasma (FFP)	Median: 0; 121 received FFPs (43.5%)	0–14	277
Cristalloids intraoperatively	Median: 6000 ml	1000–1900 ml	277
Colloids intraoperatively	Median: 0	100–3000 ml	277
Maximum intraoperative dosage of noradrenalin in µg/kg/min	Median: 7	0–80	275
Complications G0‐G5	G0‐G2: 191(68.7%); G3‐G5: 88 (31.6%)	n.a.	278
No defecation until day 3	88 (31.3%)	n.a.	278
No defecation until day 4	30 (10.8%)	n.a.	278
No defecation until day 5	12 (4.3%)	n.a.	278
Placement of a nasogastric tube in the postoperative course	23 (8.2%)	n.a.	278
Preoperative albumin	39.4 g/l	17.7–49.4 g/l	126
PFE	Median: 5400 ml	0–19, 100 ml	277
Difference between pre‐ and postoperative weight at 24 h	2.2 kg	−7.0 kg −12.6 kg	61
Difference between pre‐ and postoperative weight at day 2	4.1 kg	−7.2 kg −19 kg	166
Difference between pre‐ and postoperative weight at day 3	4.1 kg	−6.9 kg −15 kg	153
Difference between pre‐ and postoperative weight at day 4	3.1 kg	−8.8 kg −15.3 kg	187
Difference between pre‐ and postoperative weight at day 5	2.3 kg	−9.2 kg −18.4 kg	223
Day of first defecation	Median: 3	0–7	277

*Note:* Data are expressed in frequencies, medians and ranges.

Abbreviations: ASA, American Society of Anesthesiologists physical status classification; n.a.,not applicable; PCI, peritoneal cancer index.

**Table 2:** Anastomoses and anastomotic leaks.

Type of anastomosis	Number of anastomoses	Percentage	Number of leakages
Descendorectostomy	93	55.03%	13
Ileal‐ileal anastomosis	16	9.5%	1
Ileocolic anastomosis	27	15.98%	2
Transverse‐transverse colonic anastomosis	3	1.78%	0
Transeversorectostomy	9	5.33%	1
Ileorectal anastomosis	6	3.55%	1
Ascending colon‐to rectum anastomosis	6	3.55%	1
Jejunal‐jejunal anastomosis	6	3.55%	0
Jejunal‐transverse colonic anastomosis	1	0.59%	1

**Table 3:** Preoperative bowel preparation and postoperative bowel stimulation.

Preoperative bowel preparation						
Type of bowl preparation			Number of Patients		Percent of patients (%)	
None			9		3.2	
Enema only			57		20.5	
45 ml Sodium phosphate			63		22.7	
2000 ml Oralav ®			134		48.2	
2000 ml Delcoprep ®			10		3.6	
No information retrievable			5		1.8	

Postoperative bowel stimulation						
Type of bowel preparation	Number of patients without defecation on postoperative day 3 (*n* = 88)	Number of patients without defecation on postoperative day 4 (*n* = 33)		Number of patients without defecation on postoperative day 5 (*n* = 14)		Number of patients without defecation on postoperative day 6 or 7 (*n* = 9)
None	28	12		3		1
Bisacodyl	17	8		3		1
Magnesium sulfate	27	7		3		2
Metroclopramide	16	4		3		4
Enema	n.a.	1		n.a.		1
Neostigmin	n.a.	n.a.		1		n.a.
Sodium Phosphate	n.a.	1		1		n.a.

Abbreviation: n.a., not applicable.

Increasing postoperative weight gain on day 2 and 5 was associated with severe postoperative complications and anastomotic leakage. Table 4 depicts the risk of severe G3‐G5 complications and AL according to weight gain. Less weight gain within the first 5 days was associated with a shorter length of stay (median 14 d vs. median 15 d; *p*‐value: 0.03) and with an earlier first defecation (day‐2 *p*‐value: 0.01; day‐5 *p*‐value: 0.01). The immediate postoperative pH of less than 7.3 had no influence on postoperative complications (*p*‐value: 0.56) or AL (*p*‐value: 0.16). The immediate postoperative lactate of more than 1.8 mmol/l had no influence on postoperative complications (*p*‐value: 0.20) or AL (*p*‐value: 0.47). Pre‐ to postoperative hemoglobin differences of more than 4 g/dl showed an association with significantly more severe complications (*p*‐value: 0.02) without significant influence regarding ALs (*p*‐value: 0.2). Table 5 depicts further significant risk factors for postoperative complications. Preoperative albumin levels were present in 126 patients only. Low preoperative albumin levels below 30 g/dl were observed in 26 patients only without any significant effect on complications in general (*p*‐value: 1.0), severe complications (p‐value: 0.81) or ALs (*p*‐value: 0.31).

**Table 4:** Weight gain on day 2 and 5 after surgery and its association with severe G3‐G5 complications and ALs.

Weight gain and complications	Number of patients with different weight gains/Number of patients affected by G3‐G5 complications within 30 days after surgery with different weight gains at postoperative day 2	Number of patients with different weight gains/Number of patients affected by G3‐G5 complications within 30 days after surgery with different weight gains at postoperative day 5
< 3 kg > 3 kg	58 pat./G3‐G5: 10; 108 pat./G3‐G5: 29	134 pat./G3‐G5: 30; 89 pat./ G3‐G5: 34
Total number of patients	166	224
*p*‐value:	0.18	**0.02**
< 4 kg > 4 kg	76 pat./G3‐G5: 13; 90 pat/G3‐G5: 26	148 pat./G3‐35: 35; 76 pat./G3‐G5: 29
Total number of patients	166	224
*p*‐value:	**0.03**	**0.02**
< 5 kg > 5 kg	100 pat./G3‐G5: 14; 66 pat/G3‐G5: 26	159 pat./G3‐G5: 38; 65 pat/G3‐G5: 26
Total number of patients	166	224
*p*‐value:	**0.01**	**0.02**
Weight gain and AL	Number of patients with an anastomosis and number of ALs	Number of patients with an anastomosis and number of ALs
< 3 kg versus > 3 kg	22 pat/AL: 1; 42 pat/AL: 8	64 pat/AL: 6; 49 pat/AL: 11
Total number of patients with an anastomosis	64	113
*p*‐value:	0.15	0.07
< 4 kg > 4 kg	28 pat/AL: 2; 36 pat/AL: 7	72 pat/AL: 8; 40 pat/AL:9
Total number of patients with an anastomosis	64	113
*p*‐value	0.49	0.17
< 5 kg > 5 kg	35 pat/AL: 3; 29 pat/AL: 6	75 pat/AL: 9; 38 pat/AL: 8
Total number of patients with an anastomosis	64	113
*p*‐value:	0.14	0.27
< 6 kg > 6 kg	43 pat/AL: 3; 21 pat/AL: 6	84 pat/AL: 10: 29 pat/AL: 7
Total number of patients with an anastomosis	64	113
*p*‐value:	**0.021**	0.1251

**Table 5:** Risk factors for severe complications G3‐G5.

Risk factor G3‐ G5 in *n* patients/*n* patients of the entire group	*p* value
FIGO‐Stage: </= IIIB: G3‐G5 in 14/77 versus FIGO‐Stage > IIIB: G3‐G5 in 74/201	0.02
SCS: SCS </= 2: G3‐G5 in 39/165 versus SCS> 2: G3‐G5 in 49/114	0.01
Smoker: Yes: G3‐G5: in 19/69 versus No: G3‐G5 in 69/209	0.01
Performance of an anastomosis: Yes: G3‐G5 in 59/137 versus No: G3‐G5 in 29/141	0.01
Greatest pre‐ and postoperative Hb‐difference≥ 4 g/dl: G3‐ G5 in 49/125 versus < 4 g/dl: G3‐ G5 in 39/153	0.02
Age (median: 62): < median: G3‐ G5 in 38/134 versus ≥ median: G3‐G5 in 50/144	0.30
ASA Status: ASA I/II: G3‐G5 in 53/183 versus ASA III/IV: G3‐ G5 in 35/92	0.13
Duration of surgery (median 342 min): < median: G3‐G5 in 26/138 versus ≥ median: G3‐G5 in 62/140	0.00
Albumin: Albumin < 30 g/dl G3‐G5 in 7/26 versus Albumin > 30 g/dl G3‐G5 in 34/100	0.81
Age adjusted Charlston comorbidity index (AACCI): AACI 0‐1: G3‐G5 in 23/99 versus AACCI > 1: G3‐G5 in 65/189	1.00
Bowel preparation: Use of PEG: G3‐G5 in 52/147 versus Use of sodium phosphate/enema: G3‐G5 in 33/127	0.12

There was no case of renal insufficiency observed due to surgery. The association between the occurrence of an anastomotic leak respectively the occurrence of severe complications and different variables (age, smoking, duration of surgery, pre‐ to postoperative hemoglobin difference, albumin, weight difference on day 2 and day 5, FIGO‐stage, age adjusted Charleston comorbidity index, PCI, ASA Score, performance of an anastomosis, SCS and the use of PEG‐containing mechanical bowl preparation) was assessed by binary logistic regression analyses. The occurrence of an anastomotic leak showed no significant association with any variable. The occurrence of severe complications was associated with the performance of an anastomosis, and the weight gain on day 2, while PEG‐containing mechanical bowl preparation was without significant result as depicted in Table 6. The model's pseudo‐coefficient of determination (Cox& Senll *R* ^2^) was 0.1070.

**Table 6:** Multiple binary logistics regression analysis with block wise entry to investigate the association between risk factors and the occurrence of severe postoperative complications and anastomotic leakage.

Factor	*p* value
Postoperative weight gain on day 2	**0.0019**
Performance of an anastomosis	**0.0095**
PEG‐containing bowl preparation	0.1095

Table 7 shows all complications as secondary surgical events according to the MSKCC score [19].

**Table 7:** Postoperative complications listed as secondary surgical events according to the MSKCC SSE‐Score.

Type of complication according to MSKCC	Definition	Number of Cases	Percentage
G0	No complications	102	36.7%
G1	Superficial wound complications	13	4.7%
G2	Wound infection requiring superficial opening and antibiotics: 27 urinary tract infections requiring antibiotics: 17 pleural effusions requiring drainage: 14 supraventricular cardiac arrhythmia: 12 Syncope:1 Hypertension: 3 Postoperative delirium: 2 Pneumonia: 1	75	27.0%
G3	Anastomotic leakage: 8 Pancreatic fistula requiring drainage: 6 Open abdomen managed with closure and negative pressure therapy: 10 Severe postoperative paralytic ileus: 9 Intestinocutanous fistula:1 Secondary duodenal ulceration with necrosis and peritonitis:1 Hydronephrosis, secondary to lymphocele requiring drainage and ureteral stent implantation: 3 Hypertensive crisis with the need of intensive care:1 Nervus femoralis lesion:1 Cardiac ischemia requiring stent implantation: 1 Femoral artery dissection requiring subsequent thrombectomy: 1 Supraventricular arrhythmia requiring cardioversion: 1 Pneumothorax requiring a chest tube:1 Respiratory Failure: 2 Soft tissue abscess requiring drainage:1 Intraabdominal abscess requiring drainage:1 Sinusitis requiring endoscopic treatment: 1 Hemorrhage requiring surgical intervention: 2 Pleural effusion requiring chest tube evacuation: 2 Chylic leak requiring drainage: 2 Lymphcele requiring drainage: 2 Deep vein thrombosis: 1 Pulmonary embolism: 2 Pneumonia with pleural empyema: 1 Ventricular arrhythmia requiring cardioversion:1 Aspiration followed by hypoxia requiring intubation within postoperative colonoscopy before reversal of the ileostomy:1 Colitis:1 Infected pelvic hematoma requiring drainage: 1 Intraabdominal abscess requiring drainage:1	66	23.7%
G4	Anastomotic leak with peritonitis: 12 Perforated gastric ulcer: 1 Cardia ischemia with stent implantation: 3 Hypertensive crisis requiring intensive medical care:1 Nervus femoralis lesion:1 Pneumonia with pleural empyema and septic shock followed by a lung lobectomy: 1 Intestinovesicular fistulation: 1	20	7.2%
G5	Cardiac ischemia: 1 Multiple organ dysfunction syndrome: 1	2	0.72%

## 4 Discussion

In this real‐world cohort of ovarian cancer patients undergoing cytoreductive surgery, there was a significant correlation between postoperative weight gain and severe postoperative complications. Calculating the pre‐ and postoperative weight difference proved to be the least error‐prone way of assessing postoperative fluid retention. It was a reliable method to gain significant results regarding the occurrence of severe complications. Furthermore, less weight gain was associated with an earlier first defecation postoperatively.

In detail, an increase in body weight of more than 4 kg on day 2 and more than 3 kg on day 5 was associated with more severe complications. An increase of more than 6 kg on day 2 was associated with more ALs. Furthermore, multiple binary logistic regression analysis identified postoperative weight gain on day 2 and the performance of an anastomosis as the only significant predictors of severe complications. In total, we observed a median maximum gain in body weight of 4,1 kg on day 2 and 5, while others observed more than 7 kg gain in body weight considering advanced ovarian cancer patients with upper abdominal disease only [14].

Perioperative intravenous fluid managements are classified as restrictive in case of less than 1.75 liters per day, as balanced in case of 1.75 to 2.75 liters per day and liberal in case of more than 2.75 liters a day [20]. Generally, patients with a balanced perioperative fluid managements show less complications and a shorter length of stay, compared to patients who received either a liberal or a restrictive fluid management [20]. In case of colonic resections only, a body weight increase above 2.5 kg at the first postoperative day is associated with a significant increase of postoperative complications and ALs [10, 12].

An ideal fluid management provides enough blood volume to supply organs with enough oxygen to maintain all functions at a physiological level. In contrast to fluid management guidelines in colonic cancer, several key considerations must be taken into account in ovarian cancer patients. Due to capillary leak and hypoalbuminemia hemodynamic management in ovarian cancer patients, regarding the amount of crystalloids, colloids, albumin and fresh frozen plasma are different from cancer patients without peritoneal carcinomatosis [19]. Malignant ascites, driven by a VEGF production of ovarian cancer cells, induces peritoneal neo angiogenesis and vascular permeability [13, 21]. Therefore, only a liberal intraoperative fresh frozen plasma application is able to reduce fluid demands and prevent hemodynamic deterioration [22]. The ESGO guideline generally emphasizes a liberal blood and fresh frozen plasma replacement [23, 24]. Negative impacts on survival in ovarian cancer patients due to assumed immune modulatory effects, have not been observed yet [25]. Ascites is mainly protein rich and often also contains fibrinogen and fibrin to ease metastatic peritoneal implantation [19, 26]. Clinically relevant alterations in coagulation are often observed during surgery and generally managed with FFP substitution. As ascites is usually removed during surgery, patients experience a severe protein deficit with a minimum on postoperative day 2 [19]. The intravascular protein deficits further promote fluid losses to the third space due to a deterioration of the colloid‐osmotic pressure. Consequently, fluid retention occurs essentially almost always after cytoreduction for advanced ovarian cancer in the perioperative course and may easily be measured by postoperative weight gain [14].

One proposal, to overcome intravascular protein deficits are the substitution of 2 units of human albumin and 2 FFPs for the first 3 postoperative days [19]. Major concerns are an increased incidence of adverse events such as thromboembolic events [27]. Unfortunately, the first study did not include a control group and the second study collected the data of thromboembolic events in ovarian cancer patients retrospectively, no final conclusions can be drawn for a optimization of the postoperative fluid management regarding FFPs and Human albumin [19, 27].

The ERAS guideline for perioperative care in gynecologic oncology recommends no mechanical bowel preparation and a use of oral antibiotics alone in case of planned colon resections, due to the risk of preoperative dehydration [28]. This seems conclusive with our finding of high postoperative PFEs in case of PEG containing agents for mechanical bowel preparation in our cohort. Preoperative dehydration may have been the reason for extensive intraoperative fluid resuscitation resulting in high PFEs. Nevertheless, high PFEs are important to omit as they are a risk factor for severe complications, anastomotic leaks and postoperative paralytic ileus [7]. The strategy to omit a mechanical bowel preparation may lead to a reduction of intraoperative crystalloids by 1060 ml and to a reduction of the length of stay by 4 days without effect on complications, anastomotic leak or postoperative ileus in ovarian cancer patients [29]. In case of elective rectal resections the recent prospective randomized MOBILE2 trial led to the recommendation of a mechanical bowel preparation with 2 liters of PEG containing agents and oral antibiotics (neomycin and metronidazole) due to less anastomotic leaks, fewer surgical side infections and less overall complications [30].

As fluid management strategies used in colonic cancer do not directly translate to ovarian cancer patients, our data adds valuable information to improve postoperative care in ovarian cancer patients by a daily weight check to adjust fluid management daily. Apart from this, our study shows the urgent need to standardize postoperative fluid management, as fluid retention after cytoreduction is common and serves as potentially modifiable risk factor for complications, AL and postoperative paralytic ileus [14]. Despite its clinical importance, prospective randomized data on optimal fluid management protocols in ovarian cancer patients are scarce. Therefore, our findings underscore the need for prospective trials to refine the postoperative fluid management in ovarian cancer patients.

Our study has limitations such as its retrospective single center design, prone to a selection bias. The use of different preoperative mechanical bowel preparation agents may have affected intraoperative fluid management, as we observed a PFE of more than 5 liters significantly more often in the case of using PEG containing agents such as Delcoprep ® or Oralav ®. Nevertheless, our data provides cut‐offs, which may be further evaluated in prospective trials. There was no case of renal failure due to surgery. Therefore, fluid retention due to impaired renal function was largely excluded as the cause of fluid retention. Nevertheless, due to the retrospective nature of our study we had no knowledge about a possible syndrome of inappropriate antidiureses (SIAD) as some of the essential features as urinary osmolality and urinary sodium could not be collected in our cohort of patients [31]. Furthermore, we were unable to confirm albumin as significant predictor for postoperative complications as others did, most likely due to the limited number of patients for whom preoperative albumin measurements were available [32].

## 5 Conclusion

Postoperative weight gain serves as marker for fluid retention. Postoperative weight gain and the performance of an anastomosis are risk factors for postoperative severe complications in cytoreductive surgery of ovarian cancer.

## Author Contributions

E.K.E. developed the research question, M.D. performed the data acquisition, M.M. and D.K. performed the quality control of data, M.D. and M.M. performed the data analysis and interpretation, E.K.E. performed the statistical analysis, E.K.E. and L.T.P. prepared the manuscript preparation, H.M., C.S. and A.M. edited the manuscript, and all authors reviewed the manuscript and agreed on publication of the manuscript.

## Funding

The authors received no specific funding for this work.

## Conflicts of Interest

The authors declare no conflicts of interest.

## Synopsis

Weight gain due to fluid retention is an easily measurable parameter in the postoperative setting after cytoreduction for ovarian cancer. Increased weight gain postoperatively is associated with severe complications and therefore serves as a modifiable risk factor.

## Data Availability

The data that support the findings of this study are available from the corresponding author upon reasonable request.
